# Evaluating the Prevalence and Risk Factors of Schistosomiasis Amongst School-Aged Children in Low- and Middle-Income Communities: Ehlanzeni District Municipality, South Africa, 2015–2021

**DOI:** 10.3390/tropicalmed8120522

**Published:** 2023-12-17

**Authors:** Sunnieboy Lot Njikho, Vanessa Cecilia Quan, Thokozani Patrick Mbonane, Renay Helouise Van Wyk

**Affiliations:** 1 Department of Environmental Health, Faculty of Health Sciences, University of Johannesburg, Johannesburg 2001, South Africa; sunnieboyn@nicd.ac.za (S.L.N.); tmbonane@uj.ac.za (T.P.M.); 2 Public Health Surveillance and Response, National Institute for Communicable Diseases, National Health Laboratory Service, Johannesburg 2001, South Africa; vanessaq@nicd.ac.za

**Keywords:** schistosomiasis, prevalence, district municipality, disease, risk factors, low- and middle-income community, environmental factors

## Abstract

This study aimed to assess the prevalence and identify risk factors of schistosomiasis among school-aged children in low- and middle-income communities. A retrospective cross-sectional study was conducted to review patient records of school-age children. Data on gender, age, sub-district, area residing in, patient status, history of bilharzia, presence of blood in the urine, and schistosomiasis diagnoses were collected. The data were analyzed using IBM Statistical Package for the Social Sciences (SPSS) version 27. Logistic regression was employed to determine the factors associated with schistosomiasis. The overall prevalence of schistosomiasis in the study population was 75%, with higher prevalence observed among male children (89%), children aged between 10 and 14 years (59%), urban areas (51%), and rural-dominated districts, particularly Bushbuckridge (42%) and City of Mbombela (51%). Age, especially 10–14 years old (*p ˂* 0.01; 95%CI: 1.98–2.29), a history of bilharzia (*p =* 0.01; 95%CI: 1.15–1.96), and the presence of blood in urine (*p ˂* 0.01; 95%CI: 2.02–2.40) were significantly associated with schistosomiasis while being a female child was found to be a protective factor (AOR: 0.35; CI 0.35–0.41). This study underscores the importance of implementing robust screening procedures and the necessity for health education to mitigate the high prevalence of schistosomiasis and prevent its further spread.

## 1. Introduction

Schistosomiasis is a persistent infectious disease in many low- and middle-income communities (LMICs), particularly in rural and disadvantaged communities [1,2,3]. Nearly 240 million individuals worldwide are infected with schistosomiasis, and more than 700 million people reside in endemic regions [4]. It is a common tropical disease, and poverty-stricken communities, especially in Sub-Saharan Africa, are affected most adversely. In 2000, it was estimated that schistosomiasis affects almost 120 million individuals in Africa [5]. South Africa is a high middle-income country, but around 5.2 million individuals are affected by schistosomiasis, particularly among school-aged children and especially those living in poor communities such as Mpumalanga Province [6,7,8,9].

The common species of schistosomiasis in South Africa is *Schistosoma haematobium*. *Schistosoma haematobium* is responsible for causing urogenital schistosomiasis characterized by blood in urine and a previous history of bilharzia [10,11,12]. The prevalence of schistosomiasis is higher in Sub-Saharan Africa when compared to other similar tropical areas and high-income countries [5,13,14]. In 2020, Santos and colleagues reported a 30.5% prevalence amongst the Brazilian population while in Nigeria, 93.6% of urine samples tested positive for schistosomiasis [5,13]. A 2010 study in the Eastern Cape Province (South Africa) reported a 73.2% prevalence among school children from a rural community [15].

School children from rural or poor communities are more prone to schistosomiasis due to socio-economic status, environmental factors, and a lack of access to healthcare services and infrastructure [16,17,18]. Schistosomiasis is higher among school-aged children, especially boys, because of exposure to contaminated water sources during their outdoor activities such as swimming and playing in water [2,18]. Many rural areas do not have access to water infrastructure and healthcare services to ensure adequate prevention and prompt clinical diagnosis for the appropriate management and treatment of schistosomiasis [6,19].

Children who have been previously diagnosed with schistosomiasis are at risk of developing other conditions such as malnutrition, anemia, malnutrition, and learning problems [20]. Evidence also shows that long-term schistosomiasis infection can impair vital body organs (spleen, bladder, liver, lungs, and intestine) [21]. In affected areas, the World Health Organisation (WHO) advocates for the treatment of preschool children with praziquantel, which is inexpensive, easily accessible, and efficient [22]. When the treatment is started on time and repeated in childhood, it reduces, if not completely reverses, the chance of developing severe illness, even if re-infection may occur following treatment [23].

The Ehlanzeni District Municipality (EDM) is characterized by a high population living in rural areas (72%), and the majority lack access to safe, potable water supply [24]. In addition, a study reviewing prevalence between 2011 and 2018 in other South African provinces highlighted a concerning schistosomiasis prevalence in similar settings (rural areas) within KwaZulu-Natal, Eastern Cape, Mpumalanga, and Limpopo provinces [6]. Hence, this study focused on evaluating the prevalence and risk factors of schistosomiasis in a poverty-stricken district municipality (EDM) among school-aged children.

## 2. Materials and Methods

### 2.1. Study Design and Area

**Figure 1:**
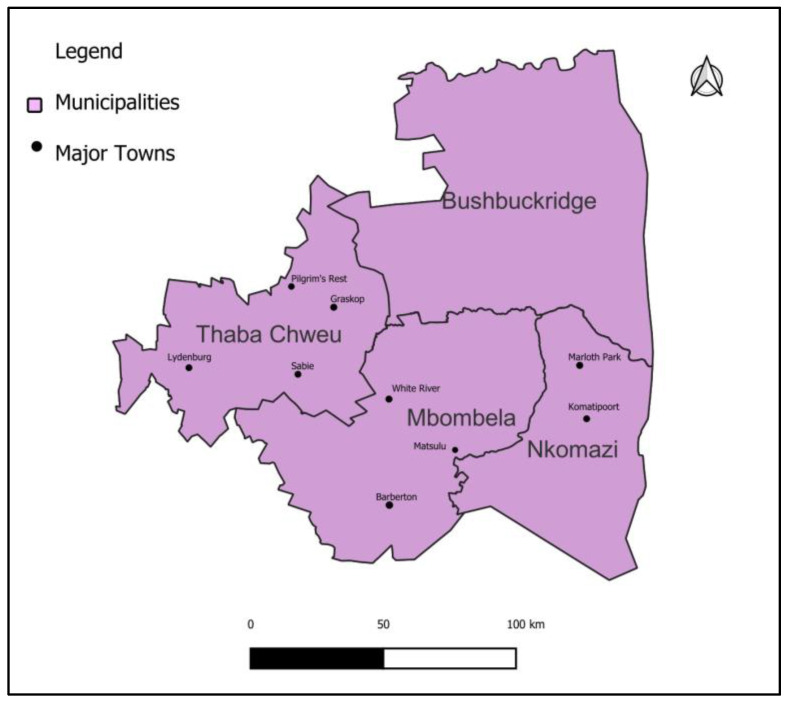
Map showing Ehlanzeni District Municipality’s sub-district.

A retrospective cross-sectional study was implemented to review secondary data of patients’ records tested for *Schistosoma haematobium ova*. The study area was one of the district municipalities in Mpumalanga, the Ehlanzeni District Municipality. The Ehlanzeni District Municipality covers 27,897.47 km^2^ and has four sub-districts in the district (Nkomazi, City of Mbombela, Bushbuckridge, and Thaba Chweu), as shown in Figure 1. The estimated population of the Ehlanzeni District Municipality was 1,856,753 people in 2019. The Ehlanzeni District Municipality is classified as a low- and middle-income area because most of the population reside in rural areas, and 77% of households survive on an average of ZAR 1600 income per month [25]. In 2022, Magagula and colleagues reported that an estimated 72% of the population of the EDM resides in rural areas [24].

The Ehlanzeni District Municipality is characterized by subtropical climatic conditions with an average annual rainfall ranging from 350 to 700 mm and an average temperature of 32 °C. It is largely flat terrain and situated at a low altitude with an estimation of a 110 to 1800 m elevation above sea level. Lastly, it is widely covered by vegetation, forest hills, and incised valleys in some sub-districts [26].

### 2.2. Study Population and Sampling

The study population was school-aged children (between 5 and 18 years of age) who had a urine test for *Schistosoma haematobium* ova in a healthcare facility within the EDM. The study included male and female children who resided in the four sub-districts in the EDM. Children younger than five years, older than 18 years, and not residing in EDM were excluded from the study. The study employed a single-stage cluster sampling approach, looking at patient records diagnosed with schistosomiasis in the Ehlanzeni District Municipality (EDM).

### 2.3. Data Collection

The data were collected by reviewing laboratory data using a record review. The following data were collected: gender, age, sub-district, patient status (whether the participant was an outpatient or hospitalized), previous diagnosis of bilharzia (in the last six months), presence of blood in the urine, and results of testing for schistosomiasis. We also collected the physical addresses of the patients (study participants) to determine the area variables (urban, semi-urban, and rural). Patient records that had missing data were not included in the study.

#### Laboratory Parasitological Test

The study focused on *Schistosoma haematobium* species as it is the most common parasite responsible for schistosomiasis in rural areas in similar settings in South Africa [19]. The urine samples were collected using 50 mL conical tubes at the facilities where the patients sought medical attention and were transported to an accredited laboratory (that conforms to international standards) within 24 h of collection. The laboratory parasitological examination was conducted by qualified and experienced laboratory technicians registered with the Medical Technology Board at the Health Professions Council of South Africa. The laboratory technicians used the urine filtration method, which involved the identification of eggs [6,27,28]. The following steps were followed: 10 mL of urine was mixed and shaken in a tube. Then, a disposable syringe was used to filter the urine through the polycarbonate filter; thereafter, a digital microscope was used to identify the presence of eggs.

### 2.4. Data Analysis

Secondary data were entered into Microsoft Excel 2021 for cleaning and coding purposes. They were moved to IBM Statistical Package for the Social Sciences (SPSS) version 27 for descriptive and inferential analyses. Duplicated samples (identified through personal identifiers) were removed from the data set, even though they had been collected for more than three weeks, as it could not be proven whether these children were properly treated on their initial diagnosis or whether they were now subsequently reinfected. Discordant results were disregarded, and cases with incomplete data on essential variables were removed. Categorical variables were used to arrange socio-demographic characteristics and schistosomiasis prevalence. These variables were presented in frequencies, percentages, and proportions. A Pearson’s correlation analysis was conducted to determine the relationship between variables, and relationships with a *p*-value of ≤0.05 were deemed to be statistically significant. To identify risk factors associated with the prevalence of schistosomiasis, logistic regression models were computed and used. The dependent variable was schistosomiasis (categorical variable: positive/negative), and the independent variables were gender (male/female), age (5–9 years old, 10–14 years old, and 15–18 years old), sub-district (Bushbuckridge/City of Mbombela/Nkomazi/Thaba Chweu), area (urban, semi-urban and rural), patient status (outpatient/hospitalized), previous history of bilharzia (yes/no), and presence of blood in urine (yes/no). A bivariate analysis was conducted, and variables that were significantly associated were included in the final model (a multivariate analysis). The relationships or associations that had a *p*-value of 0.05 were deemed statistically significant.

### 2.5. Ethical Considerations

The study received ethical approval from the University of Johannesburg, Faculty of Health Sciences, and the Research Ethics Committee, with ethical clearance number REC-1867-2022. Access to secondary data was granted through the Academic and Research wing of the (NHLS).

## 3. Result

### 3.1. Socio-Demographic Characteristics of the Study Participants

A total of 24,034 children’s samples collected from 2015 to 2021 were tested for *Schistosoma haematobium* ova in the study. Most participants were males (*n* = 19,795; 84%) and aged between 5 and 12 years (n = 13,230; 84%). More than half (n = 12,179; 51%) of the study participants were from the City of Mbombela Sub-District. There were more outpatient participants (n = 23,489; 98%) than those who were hospitalized (n = 545; 2%). Based on the clinical diagnosis, patients who had reported a previous history of bilharzia in the last six months were 351 (2%). Urine samples with the presence of blood in the urine were 4512 (19%). Table 1 shows a detailed description of the participants’ socio-demographic characteristics.

Having been previously diagnosed with bilharzia had a negative relationship with gender (*r* = −0.02, *p ˂* 0.01). The Pearson’s correlation analysis showed a negative relationship between blood in urine with gender (*r* = −0.3, *p ˂* 0.01), sub-district (*r* = −0.37, *p ˂* 0.01), and patient status (*r* = −0.05, *p ˂* 0.01). Meanwhile, it had a positive relationship with age (*r* = 0.02, *p ˂* 0.01) and area (*r* = 0.47, *p ˂* 0.01). There was no relationship (*r* = −0.01, *p =* 0.22) between the history of bilharzia and blood in urine. Table 2 shows the relationships between other variables.

### 3.2. Prevalence and Distribution of Schistosoma haematobium

There were 17,095 (71%) children that tested positive for *Schistosoma haematobium* and 29% (n = 6939) that tested negative in the study. There were more male children (n = tested positive for *Schistosoma haematobium*) compared to female children (89% vs. 11%). Children aged 10–14 years had a prevalence of 59%. The City of Mbombela had the highest prevalence according to the sub-district category. The prevalence for rural areas was 44%. Table 3 shows a detailed description of the prevalence according to gender, age, sub-district, and area classification.

### 3.3. Relationship between the Prevalence of Schistosoma haematobium and Characteristics

Female children (AOR: 0.35; CI 0.35–0.41) were less likely to be diagnosed with *Schistosoma haematobium* in the study population. Children aged between 10 and 14 years (AOR 2.12), had a history of bilharzia, and had blood in their urine were at higher odds of being diagnosed with *Schistosoma haematobium*, as shown in the multivariate logistic regression model (Table 4).

## 4. Discussion

A higher prevalence of schistosomiasis is common in rural and disadvantaged communities. According to previous studies, the prevalence of schistosomiasis ranges from 2 to 85% [5]. This study’s prevalence of schistosomiasis was 71%, which is similar to a study conducted in the Eastern Cape province, South Africa, with a prevalence of 73.2%, in 2010 [15]. A study among 4–18-year-old primary and secondary school children in Nigeria reported a prevalence of 45.6% [29]. A study in another district municipality within South Africa found a prevalence of 37.5% [28]. The higher prevalence in the study could be attributed to the study population, as the study focused on children presenting at healthcare facilities and suspected of being infected with the disease. Most studies with lower prevalence were community surveys. In addition, the areas (sub-district) with more rural communities had a higher prevalence than Bushbuckridge (42%). While a more semi-urban sub-district City of Mbombela was higher (51%), Nkomazi, a more rural sub-district, had a prevalence of 6%. This could be attributed to parents of the children seeking access to better facilities for treatment, and the patient likely having been transferred to a regional hospital from local clinics which do not have laboratory facilities or resources for urine sample collection.

Numerous previous studies have found that being a male child is a risk factor for schistosomiasis [2,30,31]. A study in Lusaka conducted among children aged 5–17 years found that male children were at a higher risk than female children to be infected with schistosomiasis [32]. This was similar to other studies in Nigeria, Gambia, and Côte d’Ivoire that found that being a male was a risk for being infected by schistosomiasis [2,29,31]. Our findings support the narrative that male children are at a higher risk. The study found that being a female child was a protective factor (AOD: 0.38, 95%CI: 0.35–0.41) to being infected with schistosomiasis. This can be attributed to girls being less involved in outdoor activities such as swimming in dams [33].

There was a significant relationship (*p* < 0.01*) between the age groups in the study. The study reported the highest cases (59%) of schistosomiasis in the age group of 10–14 years, and the logistic regression shows a link between this age and older children being diagnosed with schistosomiasis. These findings are in line with the evidence from Nigerian studies conducted in Kwara (59.4%) and Bauch State (52.8%) [29]. This study’s findings suggest that this age group could be affected because they are more adventurous and independent (as indicated by Sacolo-Gwebu and colleagues) and engage in playing in water streams such as rivers, dams, etc. [8,34].

Clinical signs and symptoms such as a history of bilharzia in the past six months and the presence of blood in urine are risk factors for schistosomiasis [2,35]. A study among Eswatini senior primary school children found a significant relationship between the prevalence of urinary schistosomiasis and the treatment of bilharzia in the last six months [36]. Furthermore, in Gambia, Joof and colleagues found that the presence of blood in urine during urine analysis was a good predisposing indicator for schistosomiasis [2]. Similar to this study, blood in urine was associated with testing positive for schistosomiasis. These are important findings for early indication of suspected schistosomiasis (medical screening) and early diagnosis for appropriate treatment. They also highlight the potential role of preventive care such as health education, environmental sampling of water sources such as dams, and access to adequate safe water sources.

The study’s strength is the sample size, which is large. The limitations are the study population which focuses on school children presenting at healthcare facilities and the study being a cross-sectional study. Therefore, the results cannot be generalized to a larger population.

## 5. Conclusions

The current study found a high prevalence of schistosomiasis in school-aged children within low- and middle-income communities, especially in rural-dominated areas. The significant risk factors were age, history of bilharzia, and presence of blood in urine, while being a male child was a risk factor. We did not find a significant relationship between schistosomiasis and area (rural, semi-urban, and urban). Based on the findings, there is a need for an interdisciplinary approach to reduce the prevalence of schistosomiasis in EDM. Tailored health education on schistosomiasis is required to be offered to children and parents, especially on the signs and symptoms, sources of infection, and contributing factors in its spread. We encourage a community survey be conducted to investigate other contributing factors in these communities.

## Figures and Tables

**Table 1:** Study participants’ socio-demographic characteristics.

Characteristics	Number (N)	Percentage
Gender		
Male	19,795	84%
Female	3628	16%
Age		
5–9 years old	4862	20%
10–14 years old	13,225	55%
15–18 years old	5947	25%
Sub-district		
Bushbuckridge	10,030	42%
City of Mbombela	12,179	51%
Nkomazi	1532	6%
Thaba Chweu	302	1%
Area		
Urban	12,170	51%
Semi-urban	1532	6%
Rural	10,332	43%
Patient Status		
Outpatient	23,489	98%
Hospitalized	545	2%
Previous history of bilharzia		
No	23,683	98%
Yes	351	2%
Presence of blood in the urine		
No	19,552	81%
Yes	4512	19%

**Table 2:** Correlation analysis between Socio-Demographic Characteristics.

Variables	Gender	Age	Sub-District	Area	Patient Status	Bilharzia History	Blood in Urine
Gender	1						
Age	0.01 * (0.74) ^#^	**1**					
Sub-district	**0.08 (** **˂0.01)**	**−0.03 (˂0.01)**	**1**				
Area	0.01 (0.72)	**0.05(˂0.01)**	**−0.67 (˂0.01)**	**1**			
Patient status	**0.12 (˂0.01)**	**−0.02 (0.02)**	**0.12 (˂0.01)**	**−0.02 (˂0.01)**	**1**		
Bilharzia history	**−0.02 (˂0.01)**	−0.01 (0.53)	0.01 (0.35)	0.01 (0.38)	−0.01 (0.23)	**1**	
Blood in urine	**−0.3 (˂0.01)**	**0.02 (˂0.01)**	**−0.37 (˂0.01)**	**0.47 (˂0.01)**	**−0.05 (˂0.01)**	−0.01 (0.22)	**1**

* Pearson correlation (*r*), ^#^ *p*-value, Bold shows that the correlation is significant (0.05).

**Table 3:** Prevalence of *Schistosoma haematobium ova* among EDM school children.

Variable	Tested Negative n (%)	Tested Positive n (%)	*p*-Value *
Gender			
Male	4983 (74%)	14,812 (89%)	<0.01 *
Female	1787 (26%)	1841 (11%)	
*Age*			
5–9 years old	2033 (29%)	2829 (17%)	<0.01 *
10–14 years old	3144 (45%)	10,081 (59%)	
15–18 years old	1762 (25%)	4185 (25%)	
Sub-district			
Bushbuckridge	2689 (39%)	7341 (43%)	<0.01 *
City of Mbombela	3334 (27%)	8836 (52%)	
Nkomazi	754 (49%)	778 (4%)	
Thaba Chweu	162 (54%)	140 (1%)	
Area			
Urban	3334 (48%)	8836 (51%)	<0.01 *
Semi-urban	754 (11%)	778 (5%)	
Rural	2851 (41%)	7481 (44%)	

* Chi-squared test used to show statistically significant association (*p =* 0.5).

**Table 4:** Regression models for risk factors of schistosomiasis in the study.

Determinants		Bivariate Model		Multivariate Model	
		COR (95% CI)	*p*-Value	AOR (95% CI)	*p*-Value
Age	5–9 years old	Ref			
	10–14 years old	2.30 (2.15–2.47)	<0.01 *	2.12 (1.98–2.29)	<0.01 *
	15–18 years old	1.70 (1.57–1.84)	<0.01 *	1.73 (1.60–1.88)	<0.01 *
Gender	Male	Ref			
	Female	0.38 (0.32–0.37)	<0.01 *	0.35 (0.35–0.41)	<0.01 *
History of bilharzia	No	Ref			
	Yes	1.48 (1.14–1.91)	<0.01 *	1.50 (1.15–1.96)	0.01 *
Blood in urine	No	Ref			
	Yes	2.16 (1.99–2.35)	<0.01 *	2.21 (2.02–2.40)	<0.01 *

Ref means reference, * *p*-value significant at 0.05.

## Data Availability

Data are available upon reasonable request and within the prescripts of the Protection of Personal Information Act (POPIAct).
